# Simultaneous and sensitive detection of *Mycobacterium tuberculosis* and SARS-CoV-2 antigens employing an electrochemical impedance spectroscopy aptasensor

**DOI:** 10.3389/fbioe.2025.1692839

**Published:** 2025-10-31

**Authors:** Zhazira Zhumabekova, Timur Elebessov, Tri Thanh Pham, Damira Kanayeva

**Affiliations:** ^1^ Ph.D. Program in Life Sciences, Department of Biology, School of Sciences and Humanities, Nazarbayev University, Astana, Kazakhstan; ^2^ Department of Biology, School of Sciences and Humanities, Nazarbayev University, Astana, Kazakhstan

**Keywords:** *Mycobacterium tuberculosis*, SARS-CoV-2, electrochemical impedance spectroscopy, aptasensor, detection, MPT64, S-glycoprotein, simultaneous

## Abstract

MPT64 and S-glycoprotein are essential biomarkers for detecting tuberculosis and coronavirus, two prevalent infectious diseases worldwide. In this study, we developed an electrochemical impedance spectroscopy (EIS)-based aptasensor fabricated to detect both target antigens simultaneously, employing a dual-platform approach on a screen-printed gold electrode (SPGE). Thiolated aptamers targeting both antigens were functionalized on the surface of the SPGE, which was then blocked with 6-mercapto-1-hexanol and assessed for the detection of target biomarkers after a 10-min incubation period. The performance was evaluated using EIS, which can detect target antigens in the range of 0.01 pg/mL to 10 pg/mL in both buffer and human serum, quantified through charge transfer resistance (*R*_ct_) values. For MPT64 and S-glycoprotein in buffer, the optimized aptasensor achieved detection limits of 0.053 pg/mL and 0.319 pg/mL, respectively. In human serum, the detection limit for MPT64 was 0.085 pg/mL, whereas it was 1.421 pg/mL for S-glycoprotein. The surface functionalization of the SPGE was confirmed through cyclic voltammetry, contact angle measurements, and atomic force microscopy. The aptasensor maintained good storage stability for up to 22 days. This label-free EIS-based aptasensor is a sensitive, selective, and reproducible platform for simultaneously detecting tuberculosis and SARS-CoV-2 biomarkers, demonstrating promising potential for clinical applications.

## Graphical Abstract

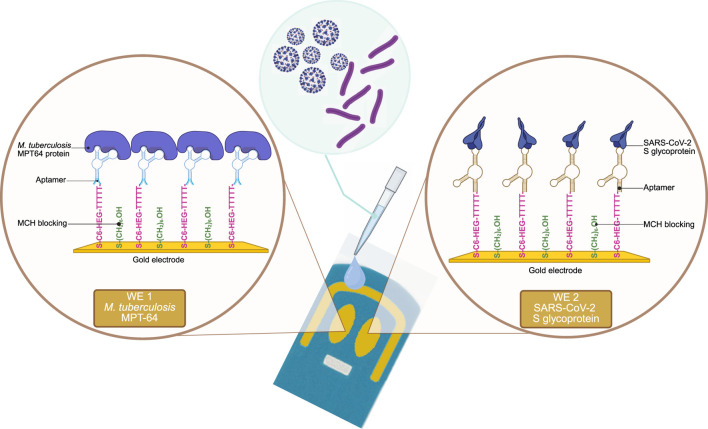

## 1 Introduction

Significant similarities exist in the pathogenesis of *Mycobacterium tuberculosis* and severe acute respiratory syndrome coronavirus 2 (SARS-CoV-2), as well as in their clinical outcomes. The lower respiratory tract is the primary site of infection for both pathogens. According to the World Health Organization (WHO, 2024), approximately 10.8 million tuberculosis (TB) cases were reported, resulting in 1.25 million deaths in 2023. In contrast, SARS-CoV-2 has led to over 760 million cases and 6.9 million deaths worldwide since December 2019 (WHO, 2023). Both diseases present similar respiratory symptoms, including cough, fever, and fatigue, which increase patient morbidity and complicate diagnosis in cases of coinfection (Cioboata et al., 2023). Recent studies have explored the connection between coinfection with SARS-CoV-2 and TB. Chen et al. (2020) proposed that TB infection increases susceptibility to SARS-CoV-2, potentially resulting in severe complications related to SARS-CoV-2 symptoms. The risk of exposure to SARS-CoV-2 may be greater for TB patients than for the general population (Kılıç et al., 2022).

Both targets can be identified using conventional polymerase chain reaction (PCR) techniques (Artik et al., 2022; Zhang et al., 2023). PCR is a sensitive and precise method for detecting targets in disease diagnosis, involving nucleic acid amplification (Canene-Adams, 2013). In the diagnosis of TB, culture of *M. tuberculosis* remains the primary gold standard method (Gholoobi et al., 2014). Isolating microorganisms through culture is essential not only for direct disease diagnosis but also for determining phenotypic drug susceptibility. Characterization through culturing is more sensitive than traditional microscopy techniques, such as acid-fast bacilli (AFB) microscopy, and is critical for drug susceptibility testing; however, it can be time-consuming and labor-intensive (Gupta et al., 2023).

Early detection of TB and SARS-CoV-2 infections is crucial for effective prevention and control, with diagnostic testing playing a key role in identifying pathogens and managing the disease. For SARS-CoV-2, various rapid detection methods have been developed that utilize serological, molecular, and nanotechnology approaches (Eftekhari et al., 2021). Label-free and sensitive electrochemical impedance spectroscopy (EIS) aptasensors (Kurmangali et al., 2022), which require minimal sample volume, along with high-throughput sequencing (Moore et al., 2020), RT-loop-mediated isothermal amplification (RT-LAMP) (Thai et al., 2004; Choi et al., 2024), and RT‒qPCR (Waggoner et al., 2020; Hanifehpour et al., 2024), are commonly used to detect viral nucleic acids and antigens, among other techniques. In addition to existing methods, multiplex fluorescence lateral flow immunoassay (LFA) (Li et al., 2025), two-channel fluorescent immunochromatographic assay (ICA) (Liu et al., 2023), CRISPR-Cas13a ICA (Wang et al., 2025), multichannel electrochemical immunoassay (EIA) (Li et al., 2021a), RT-qPCR (Karimkhani et al., 2025), radially compartmentalized paper (RCP) chip utilizing RT-LAMP (Sukumar et al., 2025), microarrays (Lee et al., 2024; Hoff et al., 2025) were developed for simultaneous detection of SARS-CoV-2 along with other common respiratory pathogens (Supplementary Table S1). Some of these diagnostic methods have limitations, including low sensitivity and specificity, high costs of instrumentation and maintenance, time consumption, the need for advanced technical skills, sample purification and preparation, and relatively slow and limited scalability (Eftekhari et al., 2021). Similarly, numerous rapid diagnostic methods have been developed to detect *M. tuberculosis*, including radiometric detection (He et al., 2011), Mycobacteria Growth Indicator Tube (MGIT) 960 and MB/BacT detection systems (Alcaide et al., 2000), PCR (Rodríguez-Lázaro et al., 2005), microarray (Malatji et al., 2023), plasmonic sensors, e.g., surface plasmon resonance (SPR) (Huang et al., 2025), high-performance plasmonic sensor based on a metal–insulator–metal (MIM) (Khodaie and Heidarzadeh, 2025), EIS-based (Agar et al., 2025; Liu et al., 2025) and immunoassays, such as enzyme-linked immunospot (ELISPOT) (Fatima, 2009), and multiplex loop-mediated isothermal amplification combined with a label-based lateral flow immunoassay (mLAMP-LFIA) (Yang et al., 2023). Compared with traditional microbial culture-based techniques, these technologies offer greater sensitivity in a shorter timeframe (He et al., 2011). However, the main drawbacks of these systems are their inability to detect pathogens rapidly and the need for expensive resources and technicians with specialized training. Therefore, developing quick, adaptable, sensitive, and specific methods to detect immunogenic proteins, such as MPT64, which is secreted by *M. tuberculosis,* is crucial. To the best of our knowledge, there are currently no data available on the development of rapid tools for the simultaneous detection of both SARS-CoV-2 and *M. tuberculosis* antigens. However, based on a literature search, some studies have conducted simultaneous detection of these targets separately, along with other antigens (Li et al., 2021b; Wang et al., 2022; Yunus et al., 2022).

In this study, we developed an EIS aptasensor to simultaneously detect two important respiratory pathogens, *M. tuberculosis* and SARS-CoV-2, one bacterial and the other viral in origin. A dual screen-printed gold electrode (SPGE) served as the transducer and was functionalized with aptamers to detect MPT64 and the S glycoprotein. This technique is label-free, sensitive, and specific, enabling the simultaneous detection of *M. tuberculosis* and SARS-CoV-2 antigens in buffer and human serum. Furthermore, this research aims to contribute to the development of rapid, accurate, and cost-effective diagnostic tools for the multiplex detection of pathogens.

## 2 Experimental

### 2.1 Reagents and materials

All aptamer sequences were custom-synthesized by Eurogentec (Belgium), and the sequences are listed in Table 1. The SARS-CoV-2 S glycoprotein (cat. no. 40592-VNAH), monkey pox virus (MPXV) A29 protein (cat. no. 40891-V08E), and Middle East respiratory syndrome-related coronavirus (MERS-CoV) S glycoprotein (cat. no. 40071-V08B1) were purchased from Sino Biological (China). The recombinant *M. tuberculosis* MPT64 protein (cat. no. AB225589) was obtained from Abcam (UK). Human serum from human male AB plasma, sourced from the United States and sterile filtered (cat. no. H4522), tris (2-carboxyethyl) phosphine hydrochloride (TCEP) (cat. no. C4706), potassium hexacyanoferrate (II) trihydrate (K_4_Fe(CN)_6_·3H_2_O) (cat. no. P3289), potassium hexacyanoferrate (III) (K_3_Fe(CN)_6_) (cat. no. 244023), phosphate-buffered saline (PBS) (cat. no. P4417), mercapto-1-hexanol (MCH) (HS(CH_2_)_6_OH) (cat. no. 725226), magnesium chloride (MgCl_2_) (cat. no. M8266), and nuclease-free water (DNase, RNase, and protease-free) (cat. no. W4502) were purchased from Sigma Aldrich. Tris-hydrochloride (Tris-HCl) (cat. no. H5123) was obtained from Promega Corporation (United States). Bovine serum albumin (BSA) (cat. no. BPE1600-100) was acquired from Fisher Scientific (UK). Sodium chloride (NaCl) (cat. no. 381659.1214) was obtained from PanReac AppliChem (Spain).

**TABLE 1:** Aptamer sequences and modifications used in the study.

No.	Aptamer	Modification at 5′	Sequence (5′ to 3′)	*K*_D_	Ref.
1	MPT64 aptamer sequence (17)	HS(CH_2_)_6_-(CH_2_CH_2_O)_6_-TT-TTT	GTC-AAC-AGT-CCA-GTT-GGG-AGG-TCG-CTT-ATA-GCT-GCT-TGT-C	8.92 nM	Sypabekova et al. (2017)
2	S glycoprotein aptamer sequence (1)	HS(CH_2_)_6_-(CH_2_CH_2_O)_6_-TT-TTT	ATC-CAG-AGT-GAC-GCA-GCA-TCG-AGT-GGC-TTG-TTT-GTA-ATG-TAG-GGT-TCC-GGT-CGT-GGG-TTG-GAC-ACG-GTG-GCT-TAG-T	6.05 nM	Liu et al. (2021)

### 2.2 Apparatus

All EIS and cyclic voltammetry (CV) measurements were performed using a µStat-i MultiX multi-channel bipotentiostat, galvanostat, and impedance analyzer (cat. no. STAT-I-MULTIX), an eight-channel boxed connector (cat. no. 4MMHCAST8), a µStat cable connector for conventional electrodes (2WE) (cat. no. 800650A, I-CABSTAT), a µStat connector for dual SPGE (cat. no. BICASTDIR), and customized dual SPGEs (cat. no. X2220BT), all of which were obtained from Metrohm DropSens (Spain). This multi-channel potentiostat was equipped with eight independent channels (nodes), allowing for simultaneous measurements and significantly increasing the study’s productivity, efficiency, and multiplexing purpose. SPGEs are produced by printing conductive inks onto ceramic substrates consisting of two working electrodes (WEs), a counter electrode (CE) made of gold, and a reference electrode (RE) made of silver. Both WEs have diameters of *d*_1_ = 0.40 cm and *d*_2_ = 0.17 cm, sharing the CE and RE located in the middle of the strip. Measurements were recorded using DropView 8400 M software v.1.05.4 (Metrohm DropSens, Spain). EIS was performed over a frequency range from 0.1 to 25 kHz using 50 measurement points and an amplitude of 10 mV. CV was conducted by scanning the potential from −0.4 to 0.6 V at a scan rate of 0.1 V/s, with a step potential of 0.001 V, and was performed in triplicate.

### 2.3 Functionalization of SPGE surfaces for the simultaneous detection of the MPT64 protein and S glycoprotein

The protocol for SPGE surface functionalization was adapted from our previously reported study by Yunussova et al. (2024) and the research conducted by Zakashansky et al. (2021), with minor modifications. Before surface functionalization, the SPGEs were cleaned with 96% ethanol and subjected to a 20-min UV-ozone treatment (ProCleaner Plus system, BioForce Nanosciences, United States). After UV exposure, the electrodes were rinsed again with ethanol. Following rinsing with nuclease-free water, the bare WEs of an SPGE were air-dried before EIS measurement in a redox couple buffer containing 5 mM ferro/ferricyanide [Fe(CN)_6_]^3-/4-^ in 10 mM PBS (pH 7.6). To reduce the 5′ends of the aptamers, 100 µM of each aptamer was dissolved in reduction buffer (TCEP) at a 1:2 volume ratio for 1 h. The aptamer-TCEP solutions were diluted in working buffer to achieve a concentration of 0.5 µM. This study employed SELEX buffer, which contains 50 mM Tris-HCl, 25 mM NaCl, and 5 mM MgCl_2_ at a pH of 7.5, as the working buffer. Therefore, unless stated otherwise, all references to the working buffer refer to the SELEX buffer. The diluted aptamer solutions were subsequently heated for 5 min at 95 °C using a Stuart block heater (cat. no. SBH130DC, UK), cooled on ice for 10 min, and allowed to return to room temperature (RT) for 5 min. Aptamers against MPT64 and S glycoprotein at a concentration of 0.5 µM were drop-cast onto the surfaces of WEs at a volume of 2.5 µL and incubated for 4 h at RT in a humid chamber. WE 1 of the SPGE was functionalized with the MPT64 aptamer, whereas WE 2 was designated for the S glycoprotein aptamer. The electrode surface was washed three times with nuclease-free water to remove unbound aptamers. After the washing step, EIS signals were measured to observe changes in impedance. The surfaces of the WEs were then blocked with a 1 mM MCH solution for 16 h at 4 °C. The stock MCH was initially prepared at a concentration of 10 mM in 98% ethanol and stored at −20 °C until further use. Before application, it was diluted with the working buffer to a final concentration of 1 mM and used immediately. A range of target MPT64 protein and S glycoprotein concentrations (from 0.01 pg/mL to 10 ng/mL), diluted in the working buffer to a volume of 2.5 µL, was incubated over each WE of the SPGE surface for 10 min at RT. The surface was rinsed with nuclease-free water between each step, and signal measurements were recorded in redox couple buffer containing 5 mM ferro/ferricyanide [Fe(CN)_6_]^3-/4-^. To test the specificity of the EIS aptasensor, the target proteins MPT64 and S-glycoprotein, as well as nontarget proteins, including the MPXV A29 protein and MERS-CoV S glycoprotein, were diluted in working buffer to a concentration of 1 pg/mL. They were incubated over the aptamer-functionalized WEs for 10 min, and the EIS signal was measured in the redox couple buffer. SELEX buffer (50 mM Tris-HCl, 25 mM NaCl, 5 mM MgCl_2_, pH 7.5) was used as the background, and all charge transfer resistance (*R*_ct_) values were adjusted by subtracting this background. Fitting errors of less than 1% were accepted for data analysis. All the measurements were carried out in triplicate, and the mean values of the replicates, standard deviations, and standard errors from the mean were used to report the results.

### 2.4 Optimization study

This study tested working buffers of 1 mM PBS (pH 7.6), 10 mM PBS (pH 7.6), and SELEX (50 mM Tris-HCl, 25 mM NaCl, 5 mM MgCl_2_, pH 7.5), and the concentrations of MPT64 protein and S glycoprotein ranged from 0.1 pg/mL to 25 pg/mL, with a corresponding aptamer concentration of 1 μM. Different concentrations of both aptamers (0.5, 1.0, 1.5, and 2.0 μM) were also evaluated against their respective target proteins at a concentration of 1 pg/mL, with an incubation time of 20 min.

The effects of various blocking methods were evaluated while detecting target proteins at a concentration of 0.075 ng/mL: 1 μM aptamer with 1 mM MCH at a 1:100 ratio in 10 mM PBS (pH 7.4), 1 μM aptamer with 1 mM MCH at a 1:100 ratio combined with 2% BSA in 10 mM PBS (pH 7.4), and 1 mM MCH diluted in SELEX (pH 7.5). All blocking conditions were incubated overnight for 16 h at 4 °C. Additionally, the influence of incubation time (10, 15, 20, and 25 min) on both targets at 1 pg/mL was analyzed using working areas of the SPGE, which were functionalized with 1 μM aptamers and blocked with 1 mM MCH diluted in the working buffer. All procedures were carried out as described in Section 2.3 “Functionalization of SPGE surfaces for the simultaneous detection of the MPT64 protein and S glycoprotein”, except for the specific conditions outlined here.

### 2.5 Simultaneous detection of the MPT64 protein and S glycoprotein in serum

MPT64 protein and S glycoprotein were spiked into commercially obtained human serum, diluted 100-fold in working buffer, at a range of concentrations (0.01 pg/mL to 10 pg/mL). After the target antigens were incubated in serum (2.5 μL) on the aptamer-functionalized WE surfaces for 10 min, the WEs were rinsed with nuclease-free water, and EIS measurements were performed in a redox couple buffer. The *R*_ct_ values of the samples were subtracted from the background values, which consisted of serum diluted 100 times in the working buffer.

### 2.6 Contact angle

After each surface functionalization step, a 1 μL drop of nuclease-free water was placed onto each WE surface using the dispensing system at a dosing rate of 1 μL/s. For this study, an optical contact angle measuring system, the OCA 15 EC (DataPhysics Instruments, Germany), which is equipped with a viewing system (camera), a stage, a dispensing system (syringe), and SCA20 (v. 5.0.15) software for calculating contact angles, was used to characterize the surface of the SPGE. Surface functionalization of WEs was carried out as described in Section 2.3 “Functionalization of SPGE surfaces for the simultaneous detection of the MPT64 protein and S glycoprotein”. 1 pg/mL target proteins (MPT64 and S glycoprotein) were applied to aptamer functionalized WE surfaces, and 1 pg/mL MPXV A29 served as a control.

### 2.7 Atomic force microscopy

The morphology of the electrode surfaces was analyzed after each functionalization step using a JPK NanoWizard 4XP atomic force microscope with a supersharp NSG30_SS tip. The tip had a spring constant of 40 N/m, a resonant frequency of 320 kHz, and a 2 nm radius of curvature. Measurements were conducted in air at room temperature in quantitative imaging mode, with a scan size of 2 × 2 μm and a resolution of 10 nm/pixel. The indentation force was set to 10 nN, with a *Z*-speed of 75 μm/s. JPK Data Processing software was used for image analysis. At least three independent samples were prepared and measured for each functionalization stage, with a minimum of 30 different regions analyzed statistically.

### 2.8 Statistical analysis

Nyquist plots were created using OriginPro 2016 Sr2 (v.b9.3.2.303 academic) (OriginLab Corporation, Northampton, MA, United States). Statistical analysis was performed with GraphPad Prism 9 (v. 9.1.0) (GraphPad Software, San Diego, CA, United States). The statistical significance of all reported results was evaluated through one-way analysis of variance (ANOVA), which is based on three biological measurements.

The percentage of *R*_ct_ change was calculated using Equation 1, yielding results with a fitting error of less than 1%.

$$\left(\left(R_{\text{protein}}\;–\;R_{\text{buffer}}\right)/R_{\text{buffer}}\right)\times 100\%$$ (1)

The limit of detection (LOD) was calculated using Equation 2:

$$\text{LOD}=3\times \left(S_{a}/b\right)$$ (2)

where *S*_a_ is the standard deviation of the *y*-intercept and *b* is the slope of the calibration curve (Tabrizi and Acedo, 2022). A *p*-value of less than 0.05 was considered significant. Nevertheless, the data were assessed using the Shapiro‒Wilk test to determine whether they were normally distributed.

## 3 Results and discussion

**SCHEME 1:**
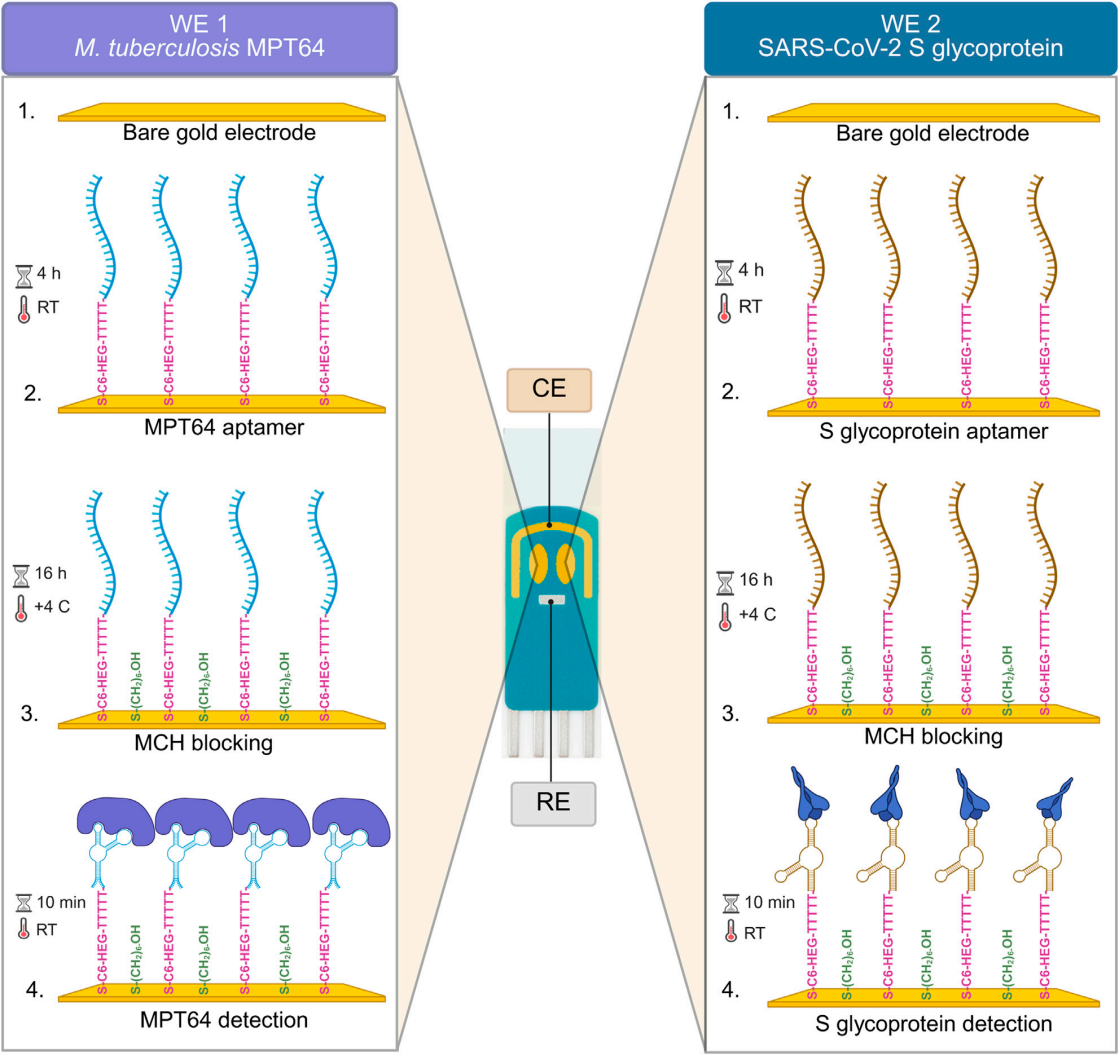
Schematic overview of the EIS aptasensor for the simultaneous detection of MPT64 and S-glycoprotein.

The experimental setup for the surface functionalization steps and the simultaneous detection of *M. tuberculosis* and SARS-CoV-2 antigens using the EIS-based aptasensor with SPGE is illustrated in Scheme 1. The morphology of the SPGE comprises two gold WEs that facilitate the functionalization of bare surfaces with two different aptamer sequences (Table 1), targeting the respective antigens, MPT64 (*M. tuberculosis*) and the S glycoprotein (SARS-CoV-2). To prevent nonspecific binding to the WEs, MCH backfilling was performed. Upon binding to the target antigens, the aptamer sequences undergo conformational changes, increasing the *R*_ct_ values.

### 3.1 Optimization of aptasensor parameters

The impact of experimental conditions on the simultaneous detection of MPT64 and S glycoprotein using SPGE with the EIS aptasensor was initially studied. The optimized parameters included the working buffer, blocking solution, aptamer concentration, and protein incubation time. Supplementary Figure S1A, B shows the results for working buffers of 1 mM PBS (pH 7.6), 10 mM PBS (pH 7.6), and SELEX (50 mM Tris-HCl, 25 mM NaCl, 5 mM MgCl_2_, pH 7.5), respectively, while target antigens ranging from 0.1 to 25 pg/mL were detected. By diluting the PBS solutions, we aimed to minimize charge screening during detection and evaluate its effect on binding efficiency. After dilution, the buffer pH was readjusted to the original value. Although 1 mM PBS showed the highest *R*_ct_ change response for detecting MPT64 and S glycoprotein, this was probably due to nonspecific adsorption or instability, as it did not demonstrate a concentration-dependent trend, making it unreliable for aptasensing. The *R*_ct_ change values for 10 mM PBS were similar to those for the SELEX buffer; however, the larger error bars indicated greater variability in the results. A concentration-dependent increase in *R*_ct_ was observed for both targets when the SELEX buffer was used. This buffer is essential in aptamer selection, where the aptamers are initially raised and selected. Our research team previously identified the aptamer sequence targeting MPT64 used in this study, which was selected using the SELEX buffer (Sypabekova et al., 2017). Similarly, Liu et al. (Liu et al., 2021) used SELEX buffer as the working buffer to select the SARS-CoV-2 aptamer 1 used in this study. This buffer also contains MgCl_2_, which enhances aptamer–protein binding (Zhang et al., 2023). Consequently, we employed the SELEX buffer as the working buffer for subsequent experiments.

In earlier work on an MPT64 aptasensor, we systematically optimized several parameters, including the aptamer-to-MCH ratio (Sypabekova et al., 2019). In that study, different ratios (1:50, 1:100, 1:200, and 1:500) were tested, and the optimal signal was achieved at 1:100, which was then used for further aptasensor development. In the present study, we extended this optimization by comparing several blocking scenarios for detecting target proteins at 0.075 ng/mL: i) aptamer:MCH at a 1:100 ratio in 10 mM PBS (pH 7.4), ii) aptamer:MCH at a 1:100 ratio with 2% BSA in 10 mM PBS (pH 7.4), and iii) 1 mM MCH in SELEX (pH 7.5) (Supplementary Figure S2A, B). The results showed that the change in *R*_ct_ was statistically significant for the 1 mM MCH solution for both targets compared with two co-immobilization strategies: aptamer:MCH (1:100) and aptamer:MCH (1:100) with 2% BSA. The observed lower *R*_ct_ change for the aptamer:MCH (1:100) ratio reflects the effect of coimmobilization of the aptamers with MCH, where MCH can displace the aptamers due to an imbalance in the ratio (Siller et al., 2020). Similarly, incubation with 2% BSA after the aptamer-MCH functionalization step did not significantly improve aptasensor performance. This suggests that the aptamers were insufficient on the electrode surface, possibly because BSA hindered their accessibility. In contrast, the use of 1 mM MCH enabled the development of a well-organized self-assembled monolayer by first functionalizing the surface with aptamers and then with MCH, thereby reducing nonspecific binding. Moreover, MCH displaces nonspecifically adsorbed portions of aptamers and promotes their upright vertical orientation, facilitating protein binding (Oberhaus et al., 2020).

**FIGURE 1:**
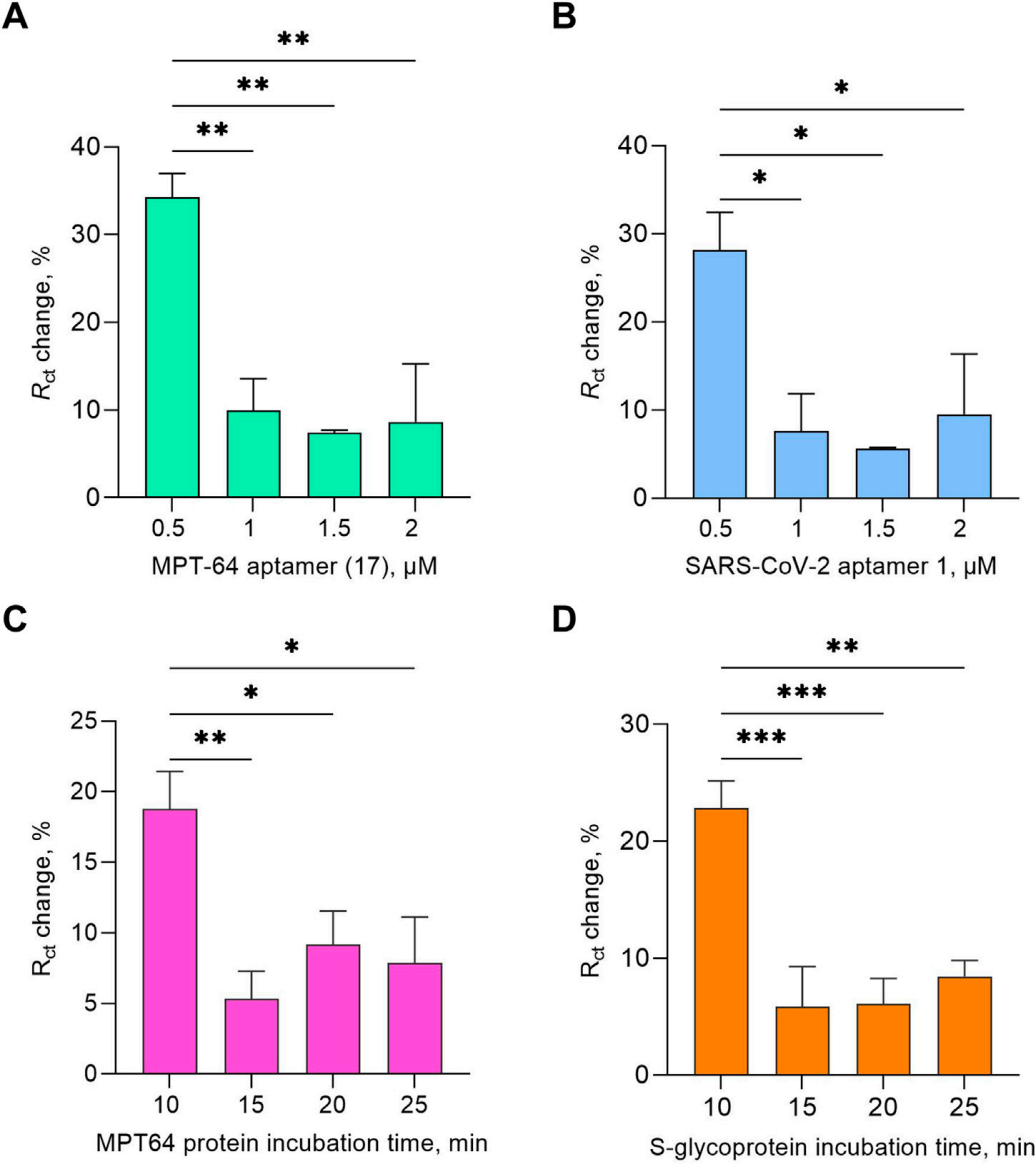
Optimization study of the EIS aptasensor testing aptamer concentration **(A,B)** and target protein incubation time **(C,D)** at a 1 pg/mL target protein concentration. The results presented in this study are averages of three biological replicates, with all the data shown as the mean values ± SEMs. Statistical significance was determined based on the *p*-value results (* *p* ≤ 0.05, ** 0.001 < *p* < 0.01, *** 0.0001 < *p* < 0.001).

Aptamers at a concentration of 0.5 μM for both target proteins showed statistically significant changes in *R*_ct_ values compared with those at concentrations of 1, 1.5, and 2 μM (Figures 1A,B). This can be explained by the aptamers forming a well-ordered monolayer at a lower concentration, allowing the optimal spacing for MCH to fill the unoccupied gold surface efficiently and facilitating favorable conformational changes in the aptamers as they bind to the target molecules. Additionally, both aptamer sequences included specific modifications, namely, SH(CH_2_)_6_-(CH_2_CH_2_O)_6_–5′-TTTTT-aptamer-3′. The thiol modification promoted strong covalent bonding, whereas the (CH_2_)_6_ spacer provided flexibility and an optimal orientation during binding with the target molecules (Zhang and Yadavalli, 2011). The HEG linker and poly(T) reduced nonspecific adsorption, minimized steric hindrance, and enhanced aptamer mobility (Zhang et al., 2025). Both aptamer sequences were specifically chosen for their high affinity and specificity for MPT64 and S-glycoprotein, which were selected using the SELEX method. They have lengths of 46 and 82 nucleotides, respectively. Their affinity was evaluated using SPR, with a dissociation constant (*K*_d_) of 8.92 nM for the MPT64 aptamer (17), which was previously selected by our research team (Sypabekova et al., 2017), and 6.05 nM for SARS-CoV-2 aptamer 1 (Liu et al., 2021), indicating strong binding (Table 1). Furthermore, the MPT64 aptamer (17) was tested on serum and sputum clinical samples from TB (+) patients, while SARS-CoV-2 aptamer 1 underwent preclinical studies using the authentic SARS-CoV-2 virus.

Additionally, the effects of incubation time (10, 15, 20, and 25 min) were tested for both targets at 1 pg/mL (Figures 1C,D). The *R*_ct_ change response yielded statistically significant results for a 10-min incubation duration for both targets. The subsequent experiments throughout the study were conducted under optimized conditions, including the use of SELEX buffer as the working buffer, 1 mM MCH as a backfilling step, 0.5 μM aptamer, and a 10 min protein incubation time.

### 3.2 Electrochemical characterization

**FIGURE 2:**
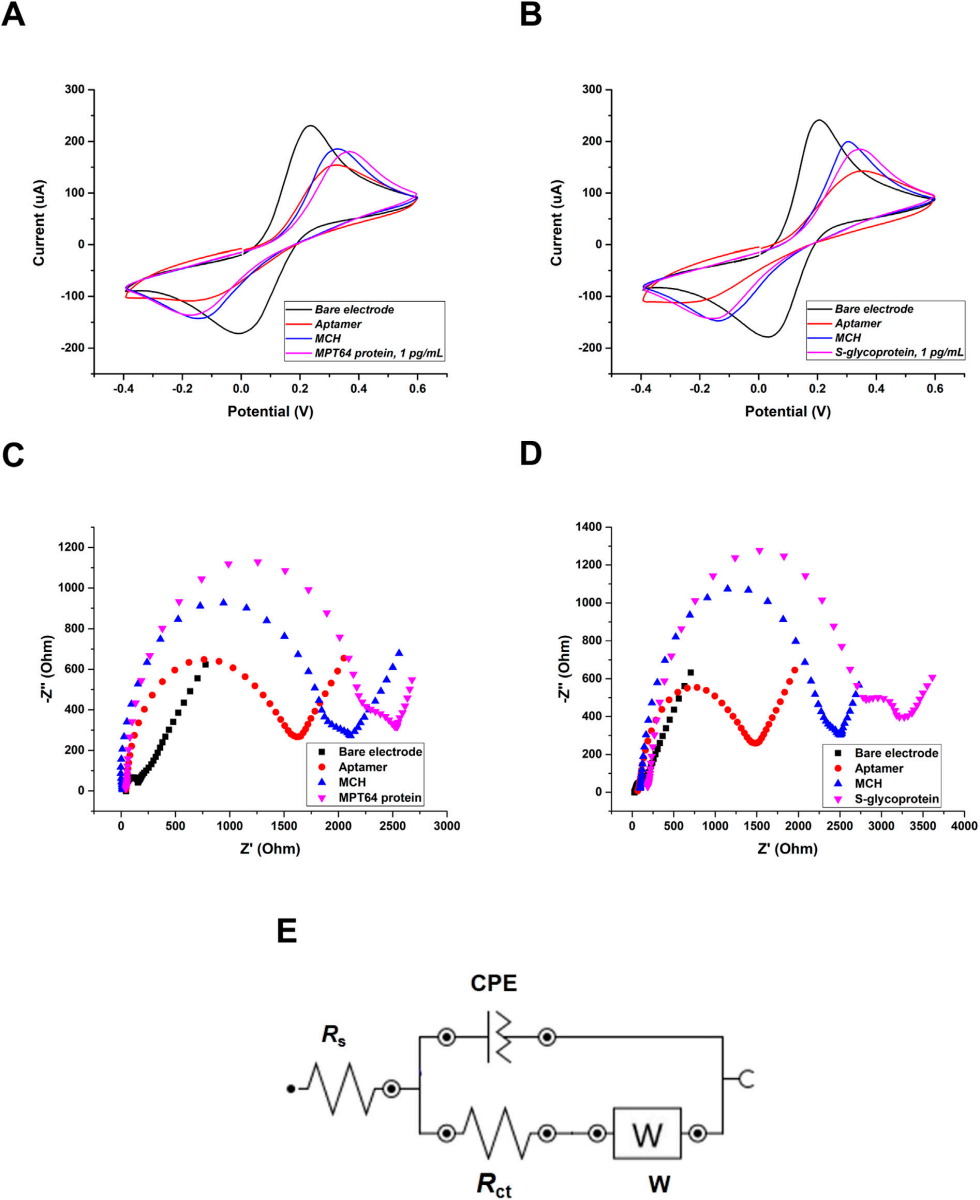
Electrochemical surface characterization of the SPGE using CV: **(A)** MPT64 and **(B)** S-glycoprotein at 1 pg/mL. Nyquist plots of stepwise characterization of the SPGE using EIS: **(C)** MPT64 and **(D)** S-glycoprotein. The Randles circuit was used to model the EIS data **(E)**, where *R*_s_ is the solution resistance, *R*_ct_ is the charge-transfer resistance, *W* is the Warburg impedance, and *CPE* is the constant phase element.

For the electrochemical measurements, CV and EIS were recorded to monitor the step-by-step modification of the surface of a dual SPGE and to evaluate the interaction between the aptamers and target proteins in the presence of a redox couple buffer containing 5 mM ferro-/ferricyanide [Fe(CN)_6_]^3-/4-^ that served as a mediator (Figure 2). CV is an important and widely applied electrochemical technique used to evaluate the reduction‒oxidation behavior of analytes and the surface characteristics of modified electrodes (Harnisch and Freguia, 2012). By measuring two major parameters, potential and current, CV provides valuable insights into electron transfer kinetics and the reversibility of electrochemical reactions (Rafiee et al., 2024). Based on the obtained CV results (Figures 2A,B), the voltammograms illustrate the gradual changes in the electrochemical response at each stage of electrode functionalization. The applied potential (from −0.4 V–0.6 V) is plotted on the *x*-axis, whereas the measured current (μA) is plotted on the *y*-axis. The bare electrode exhibited the highest current sensitivity because of its clean surface. Following the immobilization of the aptamers on the WEs, a noticeable decrease in current was observed, confirming the successful formation of a functionalized aptamer layer on the electrodes. This decrease can be attributed to steric hindrance and electrostatic repulsion created by the negatively charged phosphate backbone of the ssDNA aptamer (Yu et al., 2021). Compared with that of the aptamers, the overnight incubation of the electrode with MCH solution gradually increased, indicating successful backfilling of the unfunctionalized WE surfaces from nonspecific binding. After incubation with the target S-glycoprotein and MPT64, a decrease in current was observed, indicating successful target recognition and binding. The decrease in peak current can be explained by the steric hindrance caused by the formation of the aptamer-target complex, which further limits electron transfer at the electrode surface (Soleimani et al., 2025).

The CV results were supported by EIS and presented in the form of Nyquist plots (Figures 2C,D). In the Nyquist plot, the *x*-axis provides information about the real part of the impedance (*Z*′), which corresponds to the resistive components of the electrochemical system, including each modification and the *R*_ct_. The *y*-axis shows the negative imaginary part (-*Z*″), reflecting the capacitive behavior related to the double-layer capacitance and diffusion processes (Lazanas and Prodromidis, 2023). The bare surface showed a small semicircle (*R*_ct1_ = 71.98 Ω, *R*_ct2_ = 92.87 Ω). The functionalization with the aptamers eventually increased the semicircle, confirming the attachment of the aptamers to the electrode surface (*R*_ct1_ = 1149.35 Ω, *R*_ct2_ = 1551.78 Ω), which aligns with the obtained CV results. The semicircle gradually increased when the WEs’ surfaces were backfilled with an MCH solution (*R*_ct1_ = 1489.25 Ω, *R*_ct2_ = 1865.75 Ω). Finally, the highest *R*_ct_ was obtained after the WEs were incubated with the target antigens, which formed aptamer‒protein complexes (*R*_ct1_ = 1817.05 Ω, *R*_ct2_ = 2220.36 Ω). The gradual increase in the semicircle diameter in the high-frequency region of the Nyquist plots reflects the increase in *R*_ct,_ confirming successful surface modification (Magar et al., 2021). To fit the experimental data, the Randles circuit was employed (Figure 2E). The Randles circuit models the electrochemical interface by representing key processes with electrical components, in which *R*_s_ represents the resistance of the solution, *CPE* represents the constant phase element, *R*_ct_ represents the charge transfer resistance, and *W* represents the Warburg impedance (Magar et al., 2021). The construction of this circuit is essential for analyzing EIS data, as it allows the differentiation and quantification of various physical and chemical phenomena at the electrode interface. *R*_s_ and *W* characterize the electrolyte solution and the diffusion of the redox probe in solution. These parameters remain unaffected by electrode surface modification and the interaction of the aptamer with the protein. On the other hand, *R*_ct_ critically depends on the dielectric and insulating properties of the electrode‒electrolyte interface and can be used as a measurement parameter because it is very sensitive to changes in the electrode. *CPE* is used in an equivalent circuit instead of a simple capacitor to account for inhomogeneities and defects in the layer (Cimafonte et al., 2020). These parameters collectively enabled an accurate assessment of the aptasensor’s performance and surface functionalization.

### 3.3 Analytical performance of the EIS aptasensor for the simultaneous detection of MPT64 and S glycoprotein

**FIGURE 3:**
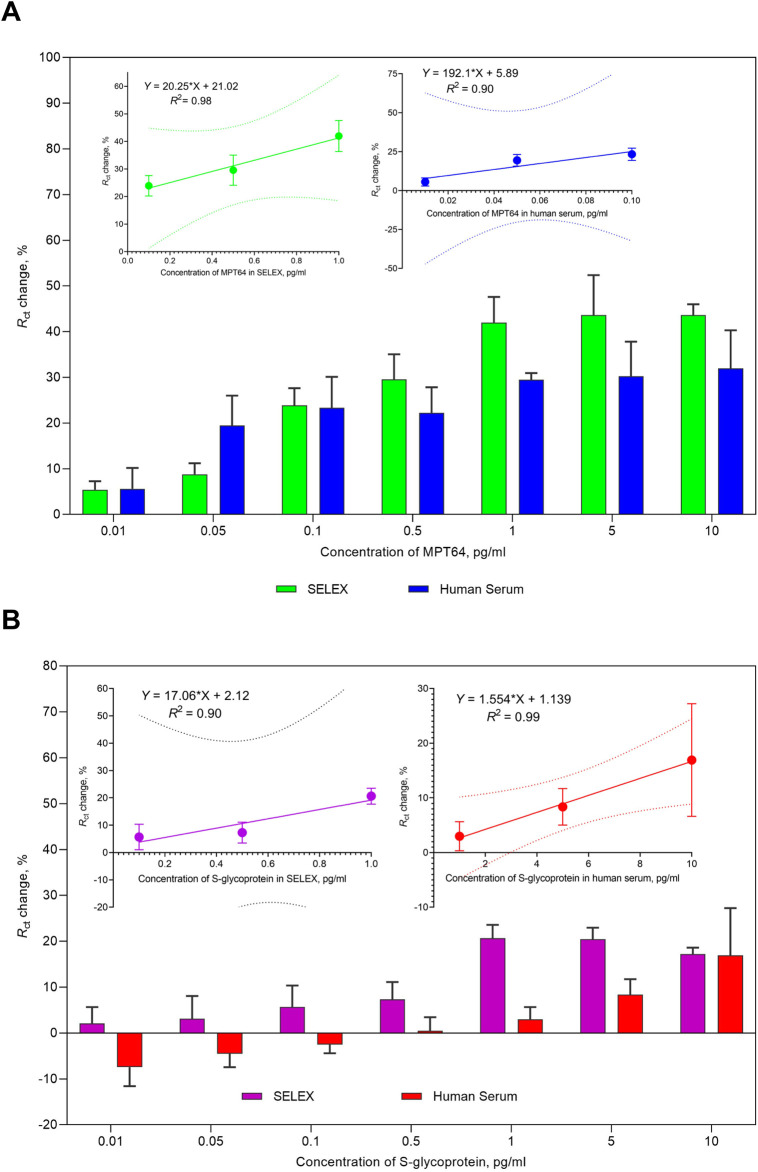
Sensitivity analysis of the EIS aptasensor for the simultaneous detection of **(A)** MPT64 and **(B)** S-glycoprotein in buffer and human serum. The insets display the linear calibration curves corresponding to the detection of **(A)** MPT64 and **(B)** S-glycoprotein in the working buffer and spiked human serum. The data are presented as the means of three independent measurements.

The sensitivity of the aptasensor was evaluated under optimized conditions (Figure 3), and the performance of the aptasensor was enhanced by reducing nonspecific interactions, maximizing the binding efficiency for the target MPT64 and S glycoprotein, and conserving reagents. Both target proteins were tested in working buffer and spiked in human serum. A change in *R*_ct_ was observed for both proteins as the MPT64 and S glycoprotein concentrations increased from 0.01 pg/mL to 10 pg/mL. At the lowest concentration of 0.01 pg/mL in the working buffer, MPT64 exhibited a 5.4% change, whereas the S glycoprotein displayed a 2.1% change in *R*_ct_. At the highest concentration of 10 pg/mL, MPT64 had a 43.6% change in *R*_ct_, whereas the S glycoprotein had a 17.2% change. Nonetheless, a plateau was observed at 1 pg/mL in the working buffer during the simultaneous detection of both proteins, indicating saturation in aptamer binding to target proteins. The insets in Figure 3A illustrate a linear correlation for MPT64 in the working buffer, with an *R* ^2^ of 98% over the concentration interval from 0.1 pg/mL to 1 pg/mL, and in human serum, with an *R* ^2^ of 90% from 0.01 pg/mL to 0.1 pg/mL. The inset in Figure 3B shows a linear correlation for the S glycoprotein in the working buffer over the concentration range from 0.1 pg/mL to 1 pg/mL, with an *R* ^2^ of 90%, and in human serum from 1 pg/mL to 10 pg/mL, with an *R* ^2^ of 99%. While detecting the target analytes simultaneously, our developed EIS aptasensor demonstrated an LOD of 0.053 pg/mL for MPT64 and a 0.319 pg/mL LOD for the S glycoprotein in SELEX buffer. Spiking the target antigens into commercially obtained human serum resulted in slightly lower sensitivity, with an LOD for MPT64 of 0.085 pg/mL and for the S glycoprotein of 1.421 pg/mL.

MPT64 and S glycoprotein are major antigens that play key roles in inducing host immune responses in their respective diseases, making them important targets for detection and diagnosis. MPT64 is a secreted protein produced by actively dividing *M. tuberculosis* and is considered an immunodominant protein (Bekmurzayeva et al., 2013). It plays a vital role in the virulence and pathogenesis of TB by modulating the body’s immune response (Kim et al., 2021). The S glycoprotein is a transmembrane protein found on the surface of SARS-CoV-2 (Banerjee et al., 2022). However, in contrast to other structural proteins, S-glycoprotein is responsible for virus attachment, fusion, and entry into the host cell by binding to receptors (Duan et al., 2020).

The reported LODs for detecting these two antigens exhibit considerable variation. Moreover, no studies have demonstrated the simultaneous detection of both, as mentioned above, although these target biomarkers have been detected in conjunction with other antigens (Supplementary Table S1). For example, Yunus et al. (2022) developed an amperometric dual aptasensor to simultaneously detect the CFP10 and MPT64 antigens of *M. tuberculosis*, utilizing sandwich aptamer‒antibody recognition elements. This biosensor employs a dual screen-printed carbon electrode (SPCE) with covalent attachment of aptamers via 4-carboxyphenyl diazonium salt, achieving LODs of 1.68 ng/mL and 1.82 ng/mL for CFP10 and MPT64, respectively. In another study, an electrochemical sandwich immunosensor with SPCEs reached a detection limit of 0.43 ng/mL in buffer (Chutichetpong et al., 2018). Although a few studies have reported slightly better LODs for MPT64 detection, for example, our research team previously reported an EIS aptasensor with an LOD of 4.1 fM in buffer using interdigitated gold electrodes (IDEs), which was further validated on serum and sputum clinical samples (Sypabekova et al., 2019). For SARS-CoV-2 S-glycoprotein detection, Lewis et al. (2021) developed an optical SPR-based aptasensor with an LOD of 0.26 nM. Wang et al. (2021) developed a two-channel fluorescent ICA to simultaneously detect SARS-CoV-2 and influenza A virus (FluA) antigens by employing silica quantum dots (QDs) and applying three layers of carboxylated QDs on a SiO_2_ surface (SiTQDs). According to the performance of this detection tool, the LODs were 5 pg/mL for SARS-CoV-2 NPs and 50 pfu/mL for FluA N1H1 antigens. Similarly, Li et al. (2021a) developed a multichannel electrochemical immunoassay (MEIA) to rapidly detect H1N1 virus and S glycoprotein, achieving LODs of 1.12 units/mL and 0.15 ng/mL, respectively. Our research team recently reported an EIS-based aptasensor for detecting S glycoprotein with an LOD of 0.4 pg/mL in buffer, which was further tested for detecting heat-inactivated variants of SARS-CoV-2, including Delta, Wuhan, and Alpha, in buffer and spiked nasal fluids with good sensitivity (Kurmangali et al., 2022). This comparison highlights that previous research has primarily focused on single biomarker detection, with no published studies on the simultaneous detection of MPT64 and S glycoprotein, which are important biomarkers for *M. tuberculosis* and SARS-CoV-2, respectively.

**FIGURE 4:**
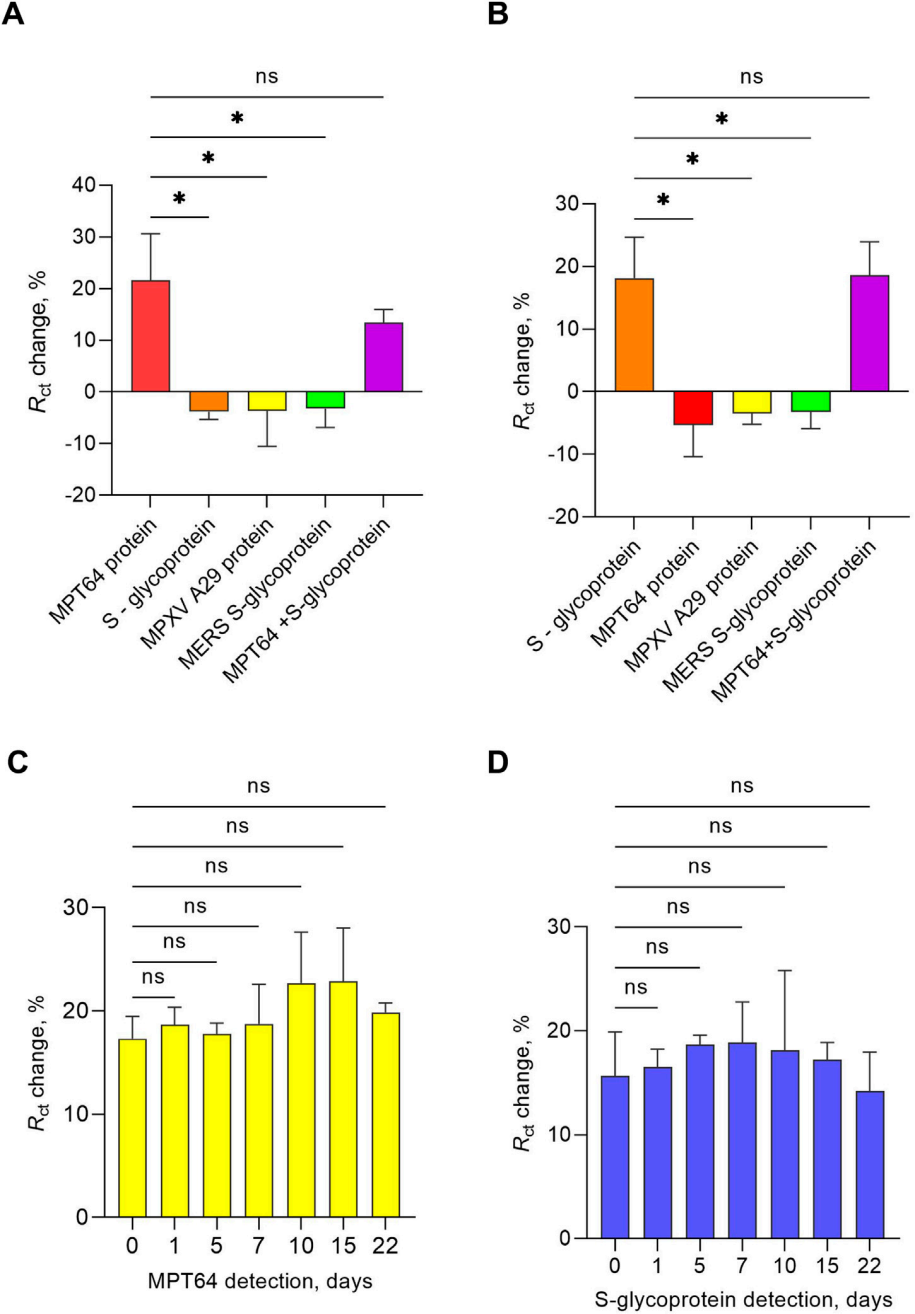
Specificity of the developed EIS aptasensor for simultaneous detection of MPT64 and S glycoprotein, with **(A)** WE 1 functionalized with the MPT64 aptamer and **(B)** WE 2 functionalized with the S glycoprotein aptamer. The target and nontarget proteins were tested at a concentration of 1 pg/mL. The data are presented as the means ± SEMs (*0.01 < *p* < 0.05). Stability of the EIS aptasensor for dual antigen detection of **(C)** MPT64 and **(D)** S-glycoprotein at a 1 pg/mL concentration at 4 °C. The data are presented as the means ± SEMs, with *n* = 3 independent replicates for each day (*ns*–nonsignificant).

The specificity of the developed EIS aptasensor for nontargeted proteins, specifically, MPXV A29, MERS-CoV S glycoprotein, the SARS-CoV-2 S glycoprotein (using the MPT64 aptamer), and MPT64 (employing the SARS-CoV-2 aptamer), was tested at a concentration of 1 pg/mL in SELEX buffer. These proteins were selected because of their significant implications for global health, primarily because of their high morbidity (Keikha et al., 2023) and their impact on healthcare systems (Waseem et al., 2022). The MPXV, which belongs to the *Orthopoxvirus* genus, has recently garnered attention worldwide following outbreaks outside endemic regions, particularly in 2022 (Karagoz et al., 2023). Recent studies suggest that the mechanism of mpox infection involves a combination of protein A29 and glycosaminoglycans on the host cell surface, indicating that A29 may serve as a biomarker for mpox detection (Sagdat et al., 2024). MERS is a highly pathogenic coronavirus classified within the *Betacoronavirus* genus (Salomon, 2024). S-glycoproteins of MERS-CoV and SARS-CoV-2 share structural similarities crucial in viral pathogenesis (Qiao et al., 2022). The EIS aptasensor exhibited statistically significant changes in the *R*_ct_ response only to the target antigens, MPT64 and S glycoprotein (SARS-CoV-2), or a combination of both targets (*p* < 0.05) (Figures 4A,B). For the MPT64 aptamer (17), the *R*_ct_ change response was 21.62% for the MPT64 protein, whereas in the presence of both target proteins, the response decreased to 13.41%. Although lower, this difference was not statistically significant and might reflect competitive binding or a steric effect under concurrent interference, without compromising detection capability (Zhang et al., 2019). For the SARS-CoV-2 aptamer (1), the *R*_ct_ change response to the S glycoprotein alone and in combination with MPT64 did not differ significantly, underscoring its high specificity. The simultaneous detection of MPT64 and S glycoprotein, along with an examination of cross-reactivity, confirmed that the aptamers employed in the developed aptasensor are specific for the target proteins.

A stability study was also conducted to ensure the reproducibility and usability of the developed EIS aptasensor for dual antigen detection. In this study, aptamer prefunctionalized SPGEs were maintained in a shaded environment at 4 °C in nuclease-free water for up to 22 days, with EIS measurements recorded in the presence of the redox couple buffer containing 5 mM ferro-/ferricyanide [Fe(CN)^6^]^3-/4-^ while detecting 1 pg/mL of both target proteins, starting from day zero. The EIS results of aptamer prefunctionalized SPGEs at 1, 5, 7, 10, 15, and 22 days were compared with those on day 0. The results in Figures 4C,D show no statistically significant changes in MPT64 and S-glycoprotein detection across different days, indicating that the developed EIS aptasensor remains stable during both short- and medium-term storage, making it suitable for applications requiring periodic measurements without the need for daily electrode functionalization.

### 3.4 SPGE surface characterization

**FIGURE 5:**
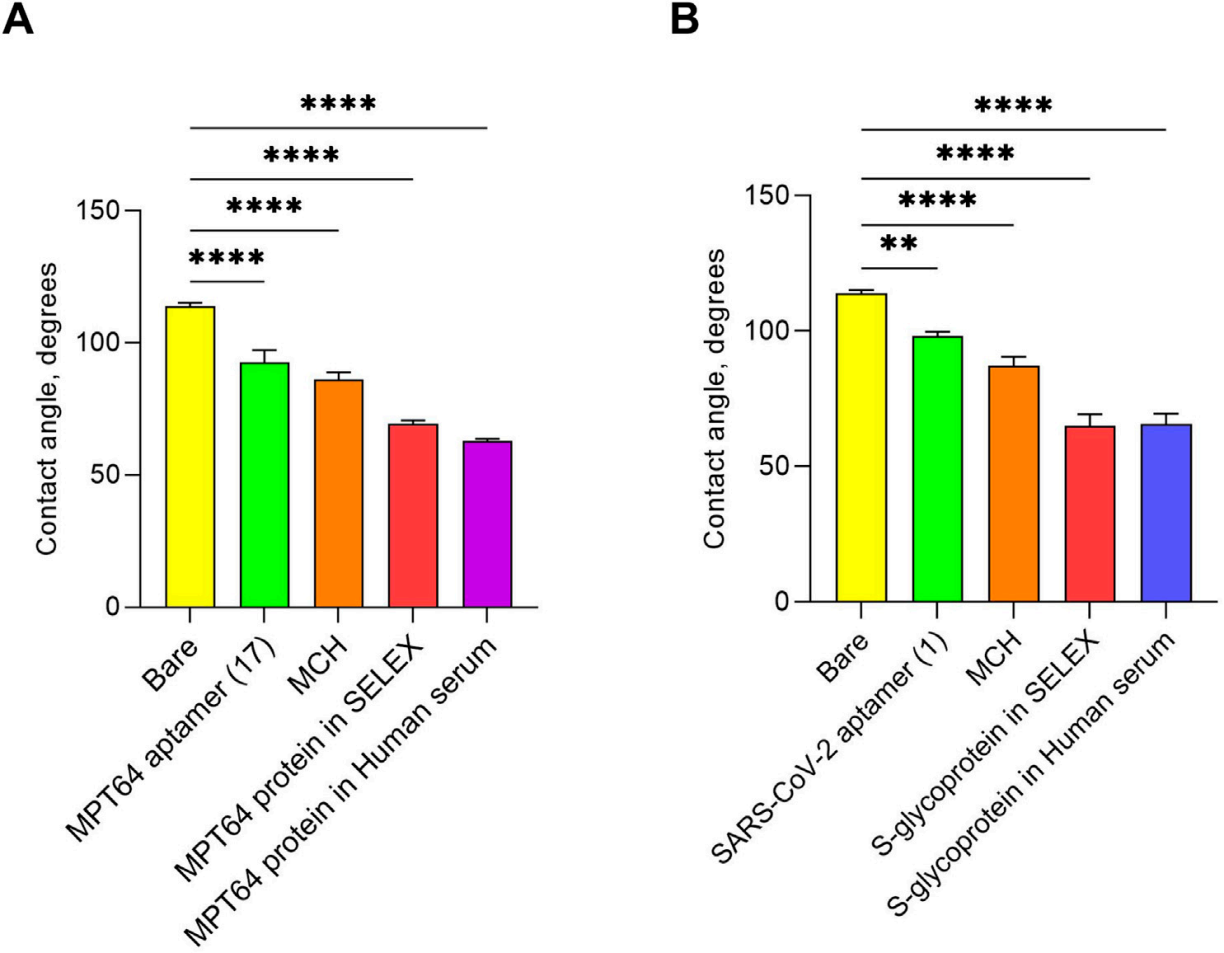
Contact angle measured at various stages of EIS aptasensor fabrication for the simultaneous detection of two antigens: **(A)** MPT64 and **(B)** S-glycoprotein. ** 0.001 < *p* < 0.01, **** 0.00001 < *p* < 0.0001 (one-way ANOVA). The experiments were conducted with three biological replicates. All the data are presented as the means ± SEMs.

The surfaces of the WEs of the SPGE were also characterized by analyzing the bare surface, aptamer-functionalized layers, MCH-blocked surfaces, and aptamer-protein binding events through contact angle measurements. Contact angle measurement is a method for characterizing surface properties by assessing a solid’s wettability by a liquid. It typically involves placing a droplet on a surface and analyzing the angle formed at the interface between the liquid, solid, and air (Akbari and Antonini, 2021). The liquid exhibited varying behaviors based on the surface modifications of the WEs (Supplementary Figure S3). For example, the contact angle measured for the deionized water drop placed on the bare SPGE was highest at 116.3°, indicating the hydrophobic nature of the electrode surface before any functionalization (Supplementary Figure S3A). SPGE is generally hydrophobic because of the natural water-repellent properties of gold itself and the electrode printing process (Frigoli et al., 2024). The screen-printing process involves the use of inks containing organic binders and solvents, contributing to its hydrophobic characteristics if it remains on the surface (Ozkan et al., 2015). The contact angles of the electrodes functionalized with the aptamers were lower than those of the bare electrodes, indicating a change in surface properties that makes the surface more hydrophilic (Hongoeb et al., 2025). After functionalization with the MPT64 aptamer (WE 1), the angle was 99.1°, whereas the SARS-CoV-2 aptamer (WE 2) displayed a value of 98.5° (Supplementary Figure S3B, E). This can be attributed to the introduction of the hydrophilic character of natural nucleotides and their successful binding (Elder and Jayaraman, 2013). Since MCH is hydrophilic (Lee et al., 2021), backfilling the aptamer-functionalized surfaces with it reduced hydrophobicity, as indicated by contact angles of 85.9° (WE 1) and 85.1° (WE 2) (Supplementary Figure S3C, F). While capturing the target antigens in the SELEX buffer, both WEs presented a significant decrease in the average contact angle values compared with those of the blocking step, indicating increased hydrophilicity (Ardalan et al., 2025; Wang et al., 2023). The contact angle for MPT64 was measured to be 68.8°, whereas that for the S-glycoprotein was 66.1° (Supplementary Figure S3D, G). Further interaction with MPT64 (Supplementary Figure S3H) and S glycoprotein (Supplementary Figure S3I), both spiked in human serum, resulted in contact angles of 63.9° and 64.6°, respectively, demonstrating specific binding to immobilized aptamers. In contrast, the control protein MPXV A29 exhibited an increase in contact angle values, 83.7° for WE 1° and 80.4° for WE 2 (Supplementary Figure S3J, K), which were similar to those of the MCH layers, 86.2° (WE 1) and 87.3° (WE 2) (Supplementary Figure S3C, F). Statistically significant data were obtained from triplicate contact angle measurements, and the average contact angle changes over the WEs’ surfaces are presented in Figure 5.

**FIGURE 6:**
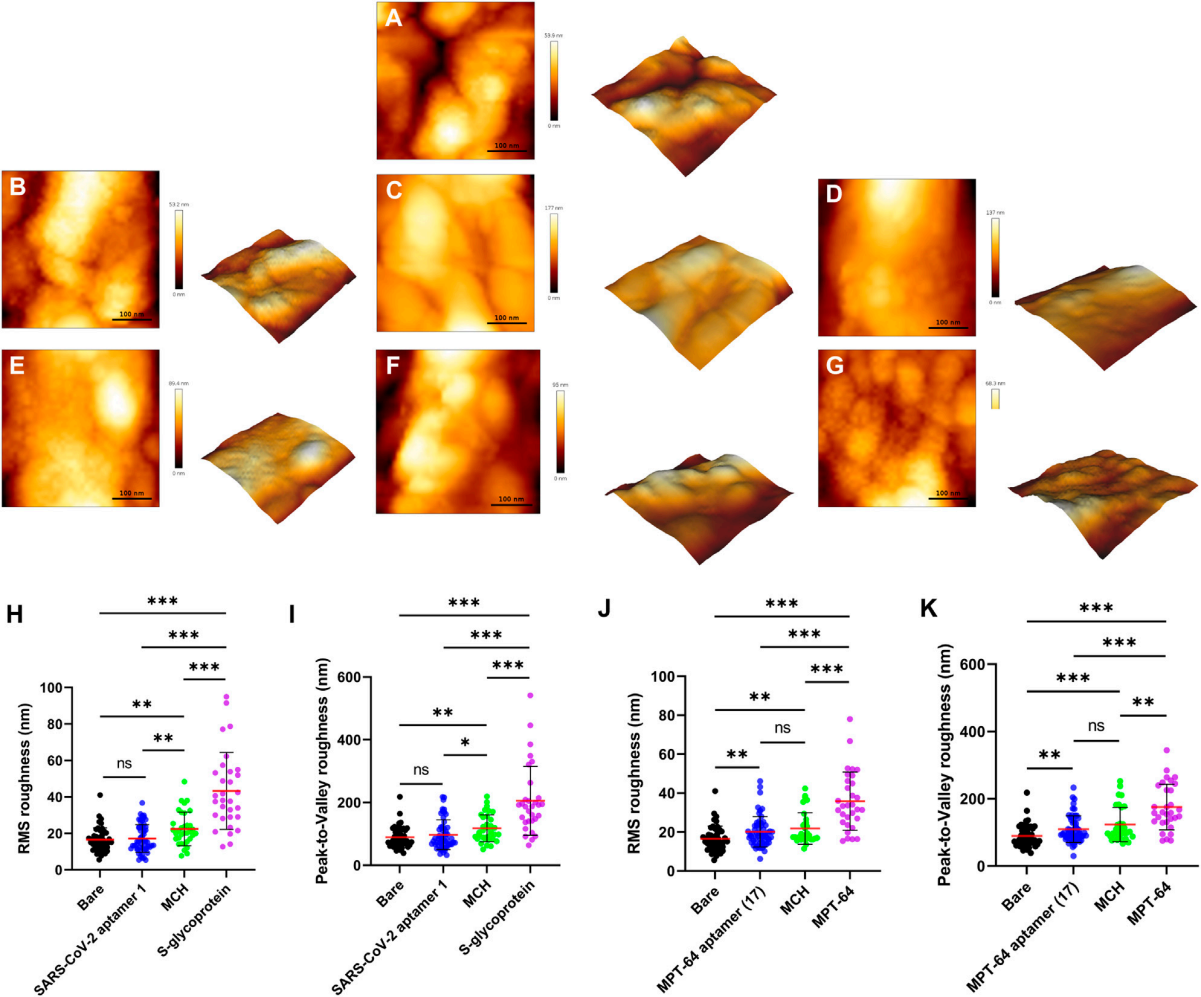
Analysis of the surface morphology of the bare and functionalized electrodes. Representative 3D images of a 0.16 μm^2^ scanned electrode surface for **(A)** a bare electrode, **(B)** functionalization with an S glycoprotein aptamer, **(C)** blocking with MCH (S glycoprotein), **(D)** binding with S-glycoprotein, **(E)** functionalization with an MPT64 aptamer, **(F)** blocking with MCH (MPT64), and **(G)** binding with MPT64. **(H)** Comparison of roughness for all functionalization steps of the S glycoprotein electrode and **(J)** the MPT64 electrode (N ≥ 31). **(I)** Comparison of height differences for all functionalization steps of the S glycoprotein electrode and **(K)** the MPT64 electrode.

Atomic force microscopy (AFM) is an ideal high-resolution imaging technique for quantifying the surface morphology of SPGE. Figures 6A–G shows the surface morphology of both WEs during all functionalization stages and their binding to the S glycoprotein and MPT64, including the bare electrode. Due to the high surface roughness of the bare electrode, as reflected in the high peak-to-valley values (Supplementary Figure S4), we had to zoom in to select the peak regions on the electrode to obtain more meaningful data for surface characterization. The root-mean-square (RMS) roughness is shown in Figures 6H,J, while the S glycoprotein and MPT64 were detected on the surface of the WEs of the SPGE during all functionalization stages. The surface of the bare electrode is relatively smooth, measuring 16.53 ± 0.95 nm (Figure 6A). The roughness of the MPT64 electrode increased to 20.11 ± 1.05 nm following aptamer functionalization (Figure 6E). However, the change in the RMS roughness of the S glycoprotein electrode was not significant (Figure 6B). Surprisingly, treatment with MCH significantly increased the roughness to 22.49 ± 1.57 nm for the WE with the S glycoprotein aptamer (Figure 6C). In contrast, the roughness of WE with the MPT64 aptamer increased to 22.17 ± 2.52 nm (Figure 6F). The roughness of the electrodes increased dramatically after treatment with the S glycoprotein or MPT64, reaching 43.26 ± 3.79 nm (Figure 6D) and 35.83 ± 2.69 nm (Figure 6G), respectively. These results suggest that the surface morphology of WEs becomes significantly rougher and more pronounced when the target proteins are successfully bound to the aptamers. Figures 6I,K illustrate the peak-to-valley roughness of the S glycoprotein (Figure 6I) and MPT64 (Figure 6K) electrodes during all functionalization stages, revealing the maximum height difference in the protein binding stage for both types of electrodes.

## 4 Conclusion

In this study, we successfully developed a novel, label-free electrochemical aptasensor for the simultaneous detection of *M. tuberculosis* and SARS-CoV-2 biomarkers. By optimizing the aptasensing conditions, the performance of the EIS aptasensor was significantly enhanced, resulting in high sensitivity and specificity, with detection limits for MPT64 of 0.053 pg/mL in buffer and 0.085 pg/mL in human serum and 0.319 pg/mL for S-glycoprotein in buffer and 1.421 pg/mL in human serum. Furthermore, the correlation between the CV, contact angle, and AFM results with the EIS data confirmed the successful characterization of each stage of functionalization and protein detection. Thus, our EIS aptasensor offers competitive detection limits and provides the benefits of simultaneous dual-analyte detection, as well as a reduced protein incubation time, making it highly suitable for practical diagnostic use. While the aptasensor demonstrated strong analytical performance in commercial serum, further evaluation using clinical samples is needed to confirm its diagnostic applicability. This will be the key direction for future research. Additionally, the possibility of integrating the developed aptasensor into point-of-care devices will also be considered. Coupling with microfluidic platforms or portable electrochemical readers could provide rapid and convenient diagnostics outside the laboratory, highlighting the potential for real-world applications.

## Data Availability

The raw data supporting the conclusions of this article will be made available by the authors, without undue reservation.
