# Supplementary material for: Simultaneous and sensitive detection of Mycobacterium tuberculosis and SARS-CoV-2 antigens employing an electrochemical impedance spectroscopy aptasensor
Source: Front Bioeng Biotechnol. 2025 Oct 31;13:1692839. doi: 10.3389/fbioe.2025.1692839 (PMC12615495; doi:10.3389/fbioe.2025.1692839)
Supplement: Supplementary file 1 [file Supplementaryfile1.docx]

Supplementary Material

**Simultaneous and sensitive detection of *Mycobacterium tuberculosis* and SARS-CoV-2 antigens employing an electrochemical impedance spectroscopy aptasensor**

**Zhazira Zhumabekova ^1^, Timur Elebessov ^1^, Tri Thanh Pham ^2^, Damira Kanayeva ^2, *^**

^1^ Ph.D. Program in Life Sciences, Department of Biology, School of Sciences and Humanities, Nazarbayev University, 53 Kabanbay batyr Avenue, Astana 010000, Kazakhstan

^2^ Department of Biology, School of Sciences and Humanities, Nazarbayev University, 53 Kabanbay batyr Avenue, Astana 010000, Kazakhstan

^*^ **Corresponding author:** Damira Kanayeva, [dkanayeva@nu.edu.kz](mailto:dkanayeva@nu.edu.kz)

**Fig. S1.** EIS aptasensor buffer optimization study. Changes in the *R*_ct_ over the surface of SPGE for **(A)** WE 1 capturing MPT64 protein (*M. tuberculosis*) and **(B)** WE 2 capturing S glycoprotein (SARS-CoV-2) across a range of concentrations while testing the effects of different working buffers: 1 mM PBS (pH 7.4), 10 mM PBS (pH 7.4), and SELEX (50 mM Tris-HCl, 25 mM NaCl, and 5 mM MgCl_2_, at pH 7.5). Data were obtained by conducting three independent experiments and are presented as means ± SEM.

**Fig. S2.** Optimization study of the blocking solution for the EIS aptasensor. Change in the *R*_ct_ over the surface of SPGE for **(A)** WE 1 capturing MPT64 protein (*M. tuberculosis*) and **(B)** WE 2 capturing the S glycoprotein (SARS-CoV-2) at a concentration of 0.075 ng/ml while testing the impact of different blocking solutions: i) aptamer:MCH at a 1:100 ratio in 10 mM PBS (pH 7.4), ii) aptamer:MCH at a 1:100 ratio with 2% BSA in 10 mM PBS (pH 7.4), and iii) 1 mM MCH in SELEX (pH 7.5). The experiments were conducted in three independent replicates, and the results are presented as means ± SEM. The reported *p*-values are the results of one-way ANOVA (*ns* – non-significant, ** 0.01 < *p* < 0.001, *** 0.001 < *p* <0.0001).

**Table S1.** Comparative table of biosensors for tuberculosis and COVID-19 detection.

| **No.** | **Method** | **Recognition element** | **Type of analyte detection** | **Sample** | **Detection limit** | | **Linear range** | | **Assay time** | | **Ref.** | |  |
| --- | --- | --- | --- | --- | --- | --- | --- | --- | --- | --- | --- | --- | --- |
| 1. | Amperometric dual aptasensor | CFP10 and MPT64 aptamers, MPT 64 and CFP 10 peroxidase-labeled secondary antibodies | dual | buffer | CFP10: 1.68 ng/ml and MPT64: 1.82 ng/ml | | CFP10: 0.5 - 100 ng/ml and MPT64: 0.75 - 250 ng/ml | | 2.5 h | | Yunus et al., 2022 | |  |
| 2. | Electrochemical sandwich immunosensor | mouse monoclonal anti-MPT64 (capture antibody), rabbit polyclonal anti-MPT 64 HRP conjugated (reporter antibody) | single | buffer, spiked in growth medium | 0.43 ng/ml in buffer | | 0.3 - 50 ng/ml and 50 - 1000 ng/ml | | 1 h 40 min | | Chutichetpong et al., 2018 | |  |
| 3. | EIS aptasensor | thiolated MPT64 aptamer | single | buffer, sputum, serum | 4.1 fM in buffer; sputum: TB(+) 100% and TB (-) 76.47%; serum: TB (+)100% and TB (-) 88.24% | | 0.1 fM - 5 nM | | 15 - 20 min | | Sypabekova et al., 2019 | |  |
| 4. | Optical LSPR -based aptasensor | S1 Aptamer, S1 Aptamer-T, N Aptamer-T | single | spiked buffer, spiked saliva | 0.25 nM | | 1 pM - 1 nM | |  | | Lewis et al., 2021 | |  |
| 5. | Two-channel fluorescent ICA | FluA monoclonal capture monoclonal antibody, SARS-CoV-2 NP monoclonal antibody | dual | inactivated SARS-CoV-2 | SARS-CoV-2 NP: 5 pg/ml and FluA N1H1: 50 pfu/ml | | SARS-CoV-2 NP: 0.01 - 100 ng/ml and H1N1: 100 - 10^5^ pfu/ml | | 15 min | | Wang et al., 2021 | |  |
| 6. | Multichannel EIA | A(H1N1) influenza virus-specific capture antibody and HRP-labeled detection antibody, SARS-CoV-2 (2019-nCoV) S glycoprotein antibody, SARS-CoV-2 (2019-nCoV) S glycoprotein antibody (HRP) | dual | serum | A(H1N1): 1.12 units/ml and SARS-CoV-2 S glycoprotein: 0.15 ng/ml | | A(H1N1): 4 - 64 unit/ml and SARS-CoV-2 S glycoprotein: 0.15 - 100 ng/ml | | - | | Li et al., 2021 | |  |
| 7. | EIS aptasensor | CoV2-RBD-1C DNA aptamer | single | buffer, heat-inactivated SARS-CoV-2 variants in buffer and spiked in human nasal fluid | S glycoprotein: 0.4 pg/ml in buffer; Delta: 6.45 ± 0.16 × 10^3^ TCID_50_/ml; Wuhan: 6.20 × 10^4^ TCID_50_/ml; Alpha: 5.32 ± 0.13 × 10^2^ TCID_50_/ml; spiked human nasal fluid: 6.45 ± 0.16 × 10^3^ TCID_50_/ml | | 0.2 - 0.8 pg/ml in buffer; inactivated SARS-CoV-2 variants: 10^1^ to 10^5^ TCID_50_/ml | | 20 min | | Kurmangali et al., 2022 | |  |
| 8. | QBs-based MF-LFA | SARS-CoV-2 capture monoclonal antibody, ADV capture monoclonal antibody, IAV capture monoclonal antibody | multiplex | spiked in nasal swab, real clinical nasal swab | SARS-CoV-2: 56 copies/ml; ADV: 120 copies/ml; IAV: 41 copies/ml | | - | | 20 min | | Li et al., 2025 | |  |
| 9. | One-Step RT-qPCR and One-Step RT-LAMP | LAMP primers | single | clinical nasal, nasopharyngeal, urine samples | RT-LAMP:  Saliva: 92 positive samples (26.9%) and 250 negative samples (73.09%).  Nasopharynx: 94 positive samples (27.4%) and 248 negative samples (72.51%).  RT-qPCR: saliva: 86 positive samples (25.1%) and 256 negative samples (74.8%).  Nasopharynx: 93 positive samples (27.1%) and 249 negative samples (72.8%). | | - | | - | | Hanifehpour et al., 2024 | |  |
| 10. | Two-channel fluorescent ICA | FluA and SARS-CoV-2 NP detecting and capturing antibodies | dual | running buffer | FluA: 2.4 pg/ml and SARS-CoV-2: 6.2 pg/ml | | 100 to 0.001 ng/ml | | 20 min | | Liu et al., 2023 | |  |
| 11. | CRISPR-Cas13a ICA | CRISPR-Cas13a | dual | - | 381.75 copies/µl | | - | | 5-10 min | | Wang et al., 2025 | |  |
| 12. | RT-qPCR | primers and probes for the HA region of inﬂuenza A, the M region of inﬂuenza B virus, and the RdRp region of SARS-COV-2 | multiplex | buffer | SARS-CoV-2: 500 copies of the virus genome, inﬂuenza A: 250 copies and inﬂuenza B: 500 copies | | 125 – 8000 copies/RXN | | - | | Karimkhani et al., 2025 | |  |
| 13. | Radially compartmentalized paper (RCP) chip utilizing RT-LAMP | primers for gene E and gene N | single and multiplex | water, spiked saliva | 10 copies/µl RNA spiked in water (single plex device) and 2040 copies/µl RNA spiked saliva samples (multiplex device) | | 10^6^ copies/μl to 10 copies/μl | | 10 min | | Sukumar et al., 2025 | |  |
| 14. | Microarray | CFP10, ESAT6, pstS1 and p24 antigens and primary antibodies | dual | buffer, human serum | CFP10, ESAT6, pstS1 and p24 antibodies LOD of 0.000954 µg/ml | | 0.000954– 250 µg/ml | | - | | Malatji et al., 2023 | |  |
| 15. | EIS-based aptasensor | MPT64 aptamer (17) and SARS-CoV-2 aptamer 1 | dual | buffer, human serum | | in buffer: 0.053 pg/ml for MPT64 and S glycoprotein 0.319 pg/ml.  Spiked in human serum: 0.085 pg/ml for MPT64, 1.421 pg/ml for S glycoprotein | | 0.01 pg/ml to 10 pg/ml in buffer and human serum | | 20 min | | This study | |

CFP10 - 10-kDa culture filtrate protein of *M. tuberculosis*; MPT64 - protein produced by the *M. tuberculosis*; HRP – horseradish peroxidase; TB – tuberculosis; EIS – electrochemical impedance spectroscopy; LSPR- localized Surface Plasmon Resonance; S – spike glycoprotein of SARS-CoV-2; N – nucleocapsid protein of SARS-CoV-2; ICA - immunochromatographic assay; FluA - Influenza A virus; NP – nucleocapsid protein; EIA - electrochemical immunoassay; H1N1 - influenza A virus subtype; QD – quantum dots; MF-LFA - multiplex ﬂuorescence lateral flow immunoassay; ADV – adenovirus; IAV - Inﬂuenza A Virus; RT-LAMP – reverse transcriptase loop-mediated isothermal amplification; RT-qPCR - reverse transcription quantitative polymerase chain reaction; HA – hemagglutinin; RdRp - RNA-dependent RNA polymerase; E – envelope; N- nucleocapsid; ESAT6 - early secreted antigenic target 6; pstS1- phosphate-binding periplasmatic protein; p24 - HIV antigen is a viral protein.


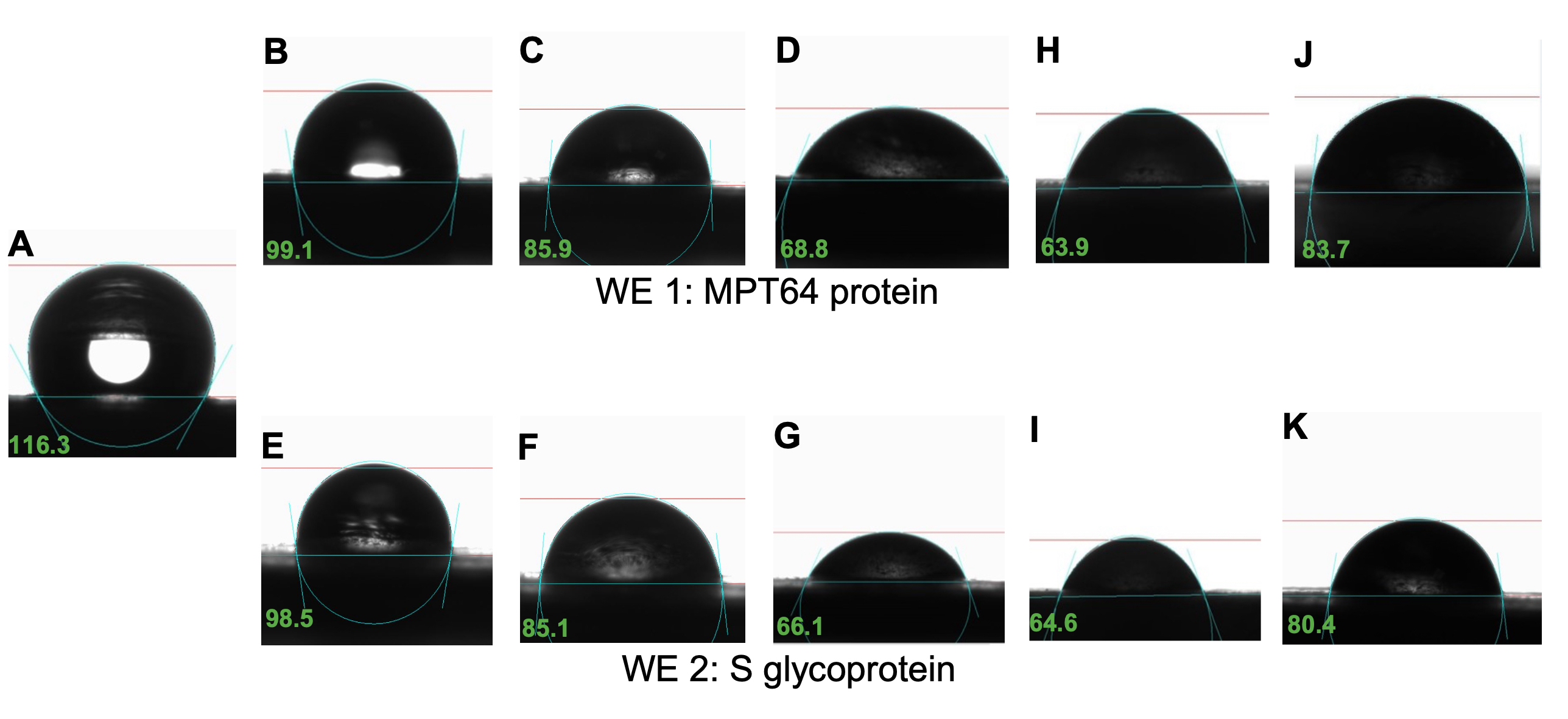


**Fig. S3.** The contact angle images illustrating WE 1 and WE 2 of the SPGE at each step of functionalization while capturing the target antigens. **(A)** bare SPGE; functionalized with **(B)** MPT64 aptamer and **(E)** S glycoprotein aptamer, **(C) and (F)** blocked with MCH; WE 1 capturing MPT64 protein **(D)** in buffer and **(H)** in human serum; WE 2 capturing S glycoprotein **(G)** in buffer and **(I)** in human serum, **(J)** and **(K)** MPXV A29 protein (control).

**Fig. S4.** Representative 3D images of a 4 μm^2^ scanned electrode surface for **(A)** bare electrode, **(B)** treatment with S glycoprotein aptamer, **(C)** blocking with MCH of viral electrode, **(D)** treatment with S-glycoprotein, **(E)** treatment with MPT64 aptamer, **(F)** blocking with MCH of bacterial electrode, **(G)** treatment with MPT64.

**References**

Agar, M., Laabei, M., Leese, H. S., and Estrela, P. (2025). Aptamer-molecularly imprinted polymer sensors for the detection of bacteria in water. doi: 10.1016/j.bios.2025.117136

Chutichetpong, P., Cheeveewattanagul, N., Srilohasin, P., Rijiravanich, P., Chaiprasert, A., and Surareungchai, W. (2018). Rapid screening drug susceptibility test in tuberculosis using sandwich electrochemical immunosensor. *Anal Chim Acta* 1025, 108–117. doi: 10.1016/J.ACA.2018.04.064

Hanifehpour, H., Ashrafi, F., Siasi, E., and Fallahi, S. (2024). Evaluation and comparison of one-step real-time PCR and one-step RT-LAMP methods for detection of SARS-CoV-2. *BMC Infect Dis* 24, 1–12. doi: 10.1186/S12879-024-09574-9/FIGURES/4

Karimkhani, S., Lotfi, E., Karamali, F., DarestaniFarahani, M., Keikha, R., and Barati, M. (2025). Development and validation of a multiplex RT-qPCR method for the simultaneous detection of influenza type A, B and SARS-COV-2 viruses. *Med Nov Technol Devices* 25, 100350. doi: 10.1016/J.MEDNTD.2025.100350

Kurmangali, A., Dukenbayev, K., and Kanayeva, D. (2022). Sensitive Detection of SARS-CoV-2 Variants Using an Electrochemical Impedance Spectroscopy Based Aptasensor. *Int J Mol Sci* 23, 13138. doi: 10.3390/IJMS232113138/S1

Lewis, T., Giroux, E., Jovic, M., and Martic-Milne, S. (2021). Localized surface plasmon resonance aptasensor for selective detection of SARS-CoV-2 S1 protein. *Analyst* 146, 7207–7217. doi: 10.1039/D1AN01458G

Li, J., Lin, R., Yang, Y., Zhao, R., Song, S., Zhou, Y., et al. (2021). Multichannel Immunosensor Platform for the Rapid Detection of SARS-CoV-2 and Influenza A(H1N1) Virus. *ACS Appl Mater Interfaces* 13, 22262–22270. doi: 10.1021/acsami.1c05770

Li, X., Zhao, C., Hou, G., Sun, Z., Liu, X., Ding, Y., et al. (2025). Simultaneously ultrasensitive and differential detection of SARS-CoV-2, adenovirus and influenza a virus using multiplex fluorescence lateral flow immunoassay. *Front Immunol* 16, 1540676. doi: 10.3389/FIMMU.2025.1540676/BIBTEX

Liu, Y., Lv, Y., Chen, W., Yang, X., Cheng, X., Rong, Z., et al. (2023). Development of a Fluorescent Immunochromatographic Assay Based on Quantum Dot-Functionalized Two-Dimensional Monolayer Ti3C2 MXene Nanoprobes for the Simultaneous Detection of Influenza A Virus and SARS-CoV-2. *ACS Appl Mater Interfaces* 15, 35872–35883. doi: 10.1021/ACSAMI.3C05424

Malatji, K., Singh, A., Thobakgale, C., and Alexandre, K. (2023). Development of a Multiplex HIV/TB Diagnostic Assay Based on the Microarray Technology. *Biosensors 2023, Vol. 13, Page 894* 13, 894. doi: 10.3390/BIOS13090894

Sukumar, P., Saleh, A., Deliorman, M., and Qasaimeh, M. A. (2025). Single-Layer Radially Compartmentalized Paper Chip (RCP-Chip) for Rapid Isothermal Multiplex Detection of SARS-CoV-2 Gene Targets. *Advanced Sensor Research* 4, 70010. doi: 10.1002/ADSR.70010

Sypabekova, M., Dukenbayev, K., Tsepke, A., Akisheva, A., Oralbayev, N., and Kanayeva, D. (2019). An aptasensor for the detection of Mycobacterium tuberculosis secreted immunogenic protein MPT64 in clinical samples towards tuberculosis detection. *Scientific Reports 2019 9:1* 9, 1–11. doi: 10.1038/s41598-019-52685-6

Wang, C., Yang, X., Zheng, S., Cheng, X., Xiao, R., Li, Q., et al. (2021). Development of an ultrasensitive fluorescent immunochromatographic assay based on multilayer quantum dot nanobead for simultaneous detection of SARS-CoV-2 antigen and influenza A virus. *Sens Actuators B Chem* 345. doi: 10.1016/j.snb.2021.130372

Wang, T., Jiang, W., Huang, Z., Yuan, Z., Chen, Z., and Lin, J. (2025). Multiplex detection of respiratory RNA viruses without amplification based on CRISPR-Cas13a immunochromatographic test strips. *Virol J* 22. doi: 10.1186/S12985-025-02765-Z

Yunus, M. H., Yusof, N. A., Abdullah, J., Sulaiman, Y., Ahmad Raston, N. H., and Md Noor, S. S. (2022). Simultaneous Amperometric Aptasensor Based on Diazonium Grafted Screen-Printed Carbon Electrode for Detection of CFP10 and MPT64 Biomarkers for Early Tuberculosis Diagnosis. *Biosensors (Basel)* 12. doi: 10.3390/bios12110996
